# Dual-Energy Computed Tomography for Evaluation of Breast Cancer Follow-Ups: Comparison of Virtual Monoenergetic Images and Iodine-Map

**DOI:** 10.3390/diagnostics12040946

**Published:** 2022-04-10

**Authors:** Jun-Xian Li, Feng-Ji Xie, Chia-Hui Chen, Kuan-Ming Chen, Chia-Jung Tsai

**Affiliations:** 1 Department of Radiology, Yuan’s General Hospital, No. 162, Chenggong 1st Rd, Lingya District, Kaohsiung City 802, Taiwan; sam76319@gmail.com (J.-X.L.); y4300_ray@yuanhosp.com.tw (F.-J.X.); 2 Department of Medical Imaging and Radiological Sciences, I-Shou University, No. 1, Section 1, Xuecheng Rd, Dashu District, Kaohsiung City 84001, Taiwan; chench2021@isu.edu.tw; 3 Department of Biomedical Imaging and Radiological Sciences, National Yang Ming Chiao Tung University, No. 155, Section 2, Linong St., Beitou District, Taipei City 112, Taiwan; gary810602@hotmail.com

**Keywords:** dual-energy computed tomography, monochromatic spectral images, iodine-map images, breast cancer

## Abstract

Differentiating tumor tissue from dense breast tissue can be difficult. Dual-energy CT (DECT) could be suitable for making diagnoses at breast cancer follow-ups. This study investigated the contrast in DECT images and iodine maps for patients with breast cancer being followed-up. Chest CT images captured in 2019 were collected. Five cases of metastatic breast cancer in the lungs were analyzed; the contrast-to-noise ratio (for breast tissue and muscle: CNR_b_ and CNR_m_, respectively), tumor-to-breast mammary gland ratio (T/B), and tumor-to-muscle ratio (T/M) were calculated. For 84 cases of no metastasis, monochromatic spectral and iodine maps were obtained to compare differences under various breast densities using the K-means algorithm. The optimal T/B, T/M, and CNR_b_ (related to mammary glands) were achieved for the 40-keV image. Conversely, CNR_m_ (related to lungs) was better for higher energy. The optimal balance was achieved at 80 keV. T/B, T/M, and CNR were excellent for iodine maps, particularly for density > 25%. In conclusion, energy of 80 keV is the parameter most suitable for observing the breast and lungs simultaneously by using monochromatic spectral images. Adding iodine mapping can be appropriate when a patient’s breast density is greater than 25%.

## 1. Introduction

According to data from the International Agency for Research on Cancer, there were 19.3 million new cancer cases and almost 10 million cancer-related deaths in 2020. Breast cancer had the highest morbidity and mortality rates for female patients. In Taiwan, breast cancer is the most common cancer in women. In 2017, the median age of cancer development was 63 years, but the median age for breast cancer development was 55 years. Therefore, the average age of patients with breast cancer is becoming lower [1,2,3]. This is due to changing diets and lifestyles, which are causing more women to not have children, not breastfeed, give birth at an older age, and have earlier menarche or later menopause. The 10-year average survival rate for patients with breast cancer was found to be 60%, the average survival rate after treatment for patients with stage 1 breast cancer was 95.5%, and the average survival rate after treatment for patients with stage 0 breast cancer was almost 100%. These data indicate that early detection of breast cancer and accurate cancer staging are crucial.

Mammography and breast ultrasound are methods commonly used to detect breast cancer. The accuracy of breast ultrasound is strongly affected by the equipment used and the operator performing the procedure; by contrast, mammography is a more standardized method. Therefore, for early-stage breast cancer that cannot be detected through palpation, mammography can be used to easily detect breast calcification. Mammography enables assessment of breast lumps detected through palpation, as well as asymptomatic breast lumps that cannot be detected through palpation. Mammography detection produces optimal results on individuals with breast atrophy and hence has excellent detection results when applied to postmenopausal women because it can detect microcalcifications and stage 0 breast cancer in which there are no symptoms, thereby reducing the breast cancer mortality rate among women aged 40–74 years. The sensitivity of mammography is affected by the stage of the patient’s menstrual cycle. Therefore, women should undergo mammography during the week after the end of their period because the sensitivity of mammography is highest and the density of breast tissues is lowest at that time [4,5,6,7].

The sensitivity of mammography ranges from 83% to 98% for fatty breasts but 48% to 64% for dense breasts [8]. In addition, recent studies have discovered that the breast cancer risk among people with dense breasts is four to six times higher than that among those with fatty breasts. In mammography, breast tissues, tumor tissues, and calcifications all appear as white spots on X-ray films, making it difficult for physicians to differentiate tumor tissues from dense breast tissues in images [9], resulting in false positive or false negative results. In addition, breast cancer can metastasize to other organs, such as the liver, lungs, and bone. Therefore, compared with conventional mammography, computed tomography (CT) is a more suitable tool for diagnosing cancer in postoperative follow-ups.

The advantages of CT include its ability to provide excellent diagnostic information and extremely clear images within short scanning times. CT has recently been upgraded to dual-energy CT (DECT). Taking advantage of the fact that different tissues attenuate X-rays to differing degrees, DECT uses dual energy spectra to obtain images with tissue characteristics or an iodinated contrast media distribution; it enables exclusion of the location difference between tissues and organs or the time difference of contrast media between tissues and organs [10,11,12]. DECT has been used to detect pathological changes that occur in metastasis of the liver, and studies have discovered that for tumors, tissues and nodules, the uniformly distributed noise in DECT spectra makes it relatively straightforward to distinguish these different media when low contrast is used [13,14]. In addition, the use of 70-keV virtual monochromatic spectral images enabled effective classification of any type of pathological change [15]. Three-phase sentinel lymph node DECT scans were used to obtain monochromatic spectral images for patients with breast cancer. The spectral slope could be used to calculate and effectively confirm whether the breast cancer had metastasized to lymph nodes; the accuracy rate was found to reach 93.5% [16]. However, few studies have used DECT for breast cancer diagnosis, and no study has researched the following-up of metastatic breast cancer.

Therefore, this study used DECT and iodine mapping to investigate contrast differences in recurrent breast cancer for breasts of various densities and thus determine whether DECT can be used to detect lesions and improve the sensitivity of postoperative follow-ups. This study had two expected outcomes. The first outcome was the optimal monochromatic spectrum, in which the contrast between mammary glands, muscles, and tumors could be identified; tumors include breast cancer and metastatic cancer in the lungs. The second outcome was the use of the K-means algorithm to calculate the breast density and investigate the contrast in the optimal monochromatic spectral images and iodine maps when different breast densities were used.

## 2. Materials and Methods

### 2.1. Patients

This study was reviewed by the Institutional Review Board (IRB) of Yuan’s General Hospital and was exempt from a full IRB review. Chest CT images obtained between January and December 2019 were collected from the Picture Archiving and Communication System of Yuan’s General Hospital; the images were captured using nonionic contrast media. The total number of cases was 2010; 1815 cases were not breast surgery cases, 80 cases were of patients who had undergone breast surgery, and 26 cases were of patients who had undergone breast implantation; hence, these cases were excluded from the current study. Of the remaining 89 cases, 5 cases were patients with metastatic breast cancer in the lungs; the 5 corresponding images were used for energy optimization. The other 84 cases were patients with breast cancer, and 80-keV monochromatic spectral images and iodine maps for each of these cases were reorganized and the data exported.

### 2.2. Image Acquisition

For all cases, scanning was performed using a GE Discovery CT750 HD scanner (Discovery CT750 HD; GE Healthcare, Milwaukee, WI, USA) in the single-source, dual-energy gemstone spectral imaging (GSI) mode. It is in one rotation by completing 80 kVp and 140 kVp instantaneous switching to collect data. Compared with the conventional multi-detector CT, it provides not only virtual monochromatic spectral images at 40–140 keV but also material decomposition images. Precontrast CT images were obtained first. A nonionic iodinated contrast agent of concentration 300 mgI/mL was administered through the antecubital vein by using a 20-gauge intravenous catheter with a power injector (Stellant D Dual Flow; Medrad, Osaka, Japan). All patients received 90 mL of contrast agent at a rate of 1 mL/s. Image processing and analysis were performed using GSI Viewer software 4.6 (GE Healthcare). The virtual monochromatic images and iodine-based material decomposition images were reviewed.

### 2.3. Image Reconstruction and Analysis

In experiment 1, the images of the five cases of metastatic breast cancer were reorganized to obtain monochromatic spectral images with the optimal outcome. Next, the reorganized CT images were exported to a computer for analysis, and the DICOM viewer was used to mark the region of interest. The virtual monochromatic spectral images for the energies 40, 50, 60, 70, 80, 90, 100, 110, 120, 130, and 140 keV were generated, and four items were marked, namely breast tumor, metastasized tumor, mammary gland, and muscle. For each item, three sections were taken and used to calculate the contrast-to-noise ratio (CNR) of the monochromatic spectral image for each patient. CNR_b_ was the CNR of breast tumor in the monochromatic spectral image and iodine map and expressed as $CNR_{b}=\frac{\left(S_{tumor}-S_{gland}\right)}{Noise_{muscle}}$. CNR_m_ was the CNR of metastasized tumor in the monochromatic spectral image and iodine map, expressed as $CNR_{m}=\frac{\left(S_{tumor}-S_{lung}\right)}{Noise_{muscle}}$. T/B was the tumor-to-breast mammary gland ratio and defined as the Hounsfield unit (HU) ratio of marked breast tumor to normal mammary glands, HU_tumor_/HU_gland_, to observe the difference between the tumor and normal mammary glands. T/M was the tumor-to-muscle ratio and is defined as the HU ratio of marked breast tumor to normal muscle tissues, HU_tumor_/HU_muscle_, to observe the difference between the tumor and normal muscle tissues. This study used analysis of variance to analyze the images obtained at energy of 40–140 keV and identify differences. ANOVA and Fisher’s least significant difference (LSD) method were employed for multiple comparisons.

The optimal monochromatic spectral image was the image obtained at 80 keV. In experiment 2, the 80-keV images of the 84 cases were reorganized. Next, the iodine map was opened to mark the areas that were exactly the same in two images. The median CNR_b_, T/B, and T/M were analyzed, and the *t* test was used for comparison.

### 2.4. Breast Density Measurement

Breast density is defined as the percentage of mammary glands in a breast. The Breast Imaging Reporting and Data System was referenced to divide breast density into four classes, namely 0–24%, 25–49%, 50–74%, and 75–100%.

The craniocaudal view was used for mammography imaging, and the K-means algorithm [17] was employed to classify the breasts on the basis of their density. The K-means method is used to cluster the whole breast image into foreground (i.e., anatomical ROIs, such as fat, muscle, and mammary gland) and background (i.e., air) clusters. Given κ=4 clusters, and the set of X grayscale pixels $X\subset {\left[-1000,\;1000\right]}^{2D}$, the κ centroid clusters C are calculated by minimizing the function u, as shown in Equation (1). Density of 0–24% was classified as fatty; 24–49%, general; 50–74%, dense; and 74–100%, extremely dense. This study hoped to evaluate the relationship between breast density and monochromatic spectral images or iodine maps.

(1) $$c=\frac{1}{\left|C_{i}\right|}\underset{x\in C_{i}}{\sum }x\;u=\underset{x\in X}{\sum }\underset{c\in C}{\min}{\left\|x-c\right\|}^{2}$$

where the centroid clusters are denoted as $C=\left\{c_{1},c_{2},\;\ldots ,\;c_{k}\right\}$.

## 3. Results

### 3.1. Experiment 1

Table 1 presents the findings for the five cases of metastatic breast cancer in the lungs; each image obtained at energies of 40–140 keV was reorganized and its T/B, T/M, CNR_b_, and CNR_m_ determined. The median T/B, T/M, and CNR_b_ for the 40-keV image were 5.04, 2.30, and 7.39, respectively; this was the best result (*p* < 0.05). Therefore, obtaining images at 40 keV can maximize the difference between tumor and tissues. However, CNR_m_ was highest for the image obtained at 140 keV; its median value was 164.58 (*p* < 0.001). This study thus discovered that a balance must be made between the greatest contrast for breast cancer and that for metastatic breast cancer in lungs.

The results of Fisher’s LSD method indicate that the optimal value of T/B was achieved for the image obtained at 40 keV; this result was significantly superior to the results when the image was obtained at 80, 90, 100, 110, 120, 130, or 140 keV (*p* = 0.03, 0.02, 0.009, 0.006, 0.004, 0.003, and 0.002, respectively). The second-best T/B value, found when the image was obtained at 50 keV, was superior to the result when the image was obtained at 110, 120, 130, or 140 keV (*p* = 0.04, 0.03, 0.02, and 0.02, respectively). The optimal T/M was achieved when the image was obtained at 40 keV; this result was superior to the results when the image was obtained at 60, 70, 80, 90, 100, 110, 120, 130, or 140 keV (*p* for 60-keV image = 0.002; all other *p* < 0.001). The T/M achieved when the image was obtained at 50 keV was superior to the result when the image was obtained at 70, 80, 90, 100, 110, 120, 130, or 140 keV (*p* for 70- and 80-keV images = 0.008 and 0.001, respectively; all other *p* < 0.001), and that for the 60-keV image was superior to the result when the image was obtained at 80, 90, 100, 110, 120, 130, or 140 keV (*p* for 80-, 90-, and 100-keV images = 0.04, 0.007, and 0.001, respectively; all other *p* < 0.001). Similarly, the T/M values for the 70-keV image was superior to those for the images obtained at 100, 110, 120, 130, and 140 keV (*p* = 0.04, 0.02, 0.007, 0.003, and 0.002, respectively). A superior T/M was found for the image obtained at 80 keV than at 130 or 140 keV (*p* = 0.04 and 0.02, respectively). The optimal CNR_b_, achieved for the image obtained at 40 keV, was superior to those achieved for the images obtained at 80, 90, 100, 110, 120, 130, and 140 keV (*p* = 0.04, 0.02, 0.01, 0.005, 0.003, 0.002, and 0.002, respectively). The CNR_b_ achieved for the image obtained at 50 keV was superior to that for the images obtained at 110, 120, 130, and 140 keV (*p* = 0.03, 0.02, 0.02, and 0.01, respectively). The optimal CNR_m_ was discovered for the image obtained at 140 keV; the differences between this CNR_m_ and the results for the images obtained at 40, 50, 60, 70, and 80 keV were significant (*p* < 0.001, <0.001, <0.001, 0.001, and 0.01, respectively). The CNR_m_ value achieved for the image obtained at 130 keV was superior to the result when the image was obtained at 40, 50, 60, 70, or 80 keV (*p* < 0.001, <0.001, <0.001, 0.003, and 0.02, respectively. The CNR_m_ value calculated for the image obtained at 120 keV was superior to that for the images obtained at 40, 50, 60, and 70 keV (*p* < 0.001, <0.001, 0.001, and 0.009, respectively). The CNR_m_ value for the image obtained at 110 keV was superior to the result for the images obtained at 40, 50, 60, and 70 keV (*p* < 0.001, <0.001, 0.004, and 0.04, respectively). The CNR_m_ value achieved when the image was obtained at 100 keV was superior to that when the image was obtained at 40, 50, or 60 keV (*p* < 0.001, 0.001, and 0.008, respectively). The CNR_m_ value achieved for the image obtained at 90 keV was superior to the result for the images obtained at 40, 50, and 60 keV (*p* < 0.001, 0.004, and 0.03, respectively). The CNR_m_ value determined for the image obtained at 80 keV was superior to that for the images obtained at 40 and 50 keV (*p* = 0.003 and 0.02, respectively). Finally, the CNR_m_ value achieved for the image obtained at 70 keV was superior to the result for the image obtained at 40 keV (*p* = 0.02).

**Figure 1:**
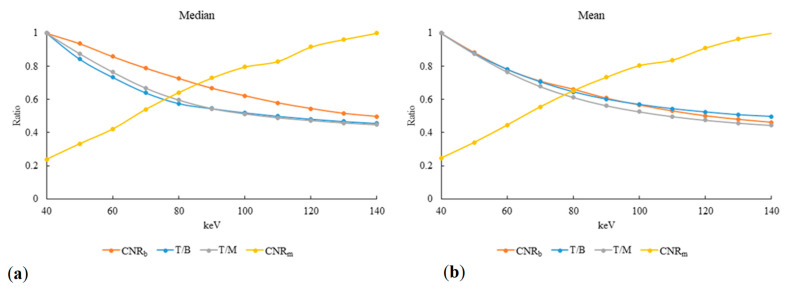
Standardized values of T/B (blue), T/M (gray), CNR_b_ (orange), and CNR_m_ (yellow) for images obtained at 40–140 keV: (**a**) median and (**b**) mean.

T/B, T/M, and CNR_b_ are related to the mammary gland, and their optimal values were achieved for the image obtained at 40 keV; as the energy was increased, the performance decreased. By contrast, CNR_m_ is related to the lungs, and the worst CNR_m_ value was attained for the image obtained at 40 keV, but CNR_m_ increased as the energy was increased. Therefore, a balance must be made to achieve the optimal result. The T/B, T/M, CNR_b_, and CNR_m_ data were standardized by selecting the maximum value as 1 and dividing the other data by the maximum value; the normalization results are presented in Figure 1. The curves of the median and mean values intersected approximately for an energy of 80 keV. Therefore, 80 keV was used as the optimal energy parameter.

### 3.2. Experiment 2

In this part of the study, the images of the 84 cases were analyzed in Table 2. In the iodine maps, the average and maximum values of T/B, T/M, and CNR_b_ were higher than those obtained from the 80-keV monochromatic spectral images (*p* = 0.002 and <0.001, *p* < 0.001 and <0.001, and *p* < 0.001 and <0.001, respectively).

Next, the K-means algorithm was used to classify the images on the basis of the four classes of breast density. The results revealed that 13 images corresponded to density in the first class (0–24%; Table 3), 59 to the second class (25–49%; Table 4), 12 to the third class (50–74%; Table 5), and 0 to the fourth class (75–100%). This study discovered that for fatty breasts, the iodine maps produced superior results to the monochromatic spectral images; however, the average T/B and T/M were not significantly different. For breasts of general or high density, the average T/B, T/M, and CNR_b_ obtained from the iodine maps were significantly higher than those obtained from the monochromatic spectral images, and as the density increased, the average and maximum values of T/B, T/M, and CNR_b_ from the iodine maps and monochromatic spectral images also increased. Therefore, contrast may become sharper when breast density is higher.

## 4. Discussion

In 2020, Okada et al. [2] investigated the optimal parameter for monochromatic spectral images of mammary glands and discovered that 40-keV images had the greatest contrast in objective analysis. The present study also discovered that the optimal T/B, T/M, and CNR_b_ were achieved for the images obtained at 40 keV. Khan et al. observed the lungs using conventional CT and DECT and reorganized monochromatic spectral images to investigate tuberculosis. Their results indicated that the monochromatic spectral images obtained at 140 keV were superior to those obtained at 80 keV and that the combination of the two types of images achieved superior results to conventional CT images [18]. The present study discovered that 140-keV images are suitable for observation of the lungs because the lungs mostly comprise air, and the best CNR_m_ results were attained for these images. In conclusion, this study focused on the follow-up of breast cancer—that is, the detection of recurrent breast cancer—and assessed the quality of images. The mammary glands and lungs, where cancer cells can metastasize, were investigated. Breast CT and lung CT are usually performed together clinically; hence, this study aimed to identify the parameter with which the optimal identification can be made. The analysis results were standardized, and 80-keV energy was discovered to be the parameter most suitable for observing the breast and lungs simultaneously (Figure 1). Other studies have also indicated that 80 keV is the optimal parameter for the observation of soft tissue images after subjective factors were included [2,18].

Gao et al. [19] used iodine-based and water-based images to identify cirrhosis nodules and malignant tumors. Compared with normal tissues, cirrhosis nodules contain little moisture, whereas malignant tumors have high levels of iodine. Therefore, the weak signals in water-based images can be used to identify cirrhosis nodules, and the strong signals in iodine-based images can be used to identify malignant tumors and mark the tumors with obscure edges. The present study did not use water-based images because malignant mammary glands do not have different moisture levels to normal mammary glands. However, the level of iodine in breast tumors is higher than that in normal mammary glands; hence, iodine maps were used to conduct the research. Water-based images can be used in the future to identify the edges of metastatic tumors.

**Figure 2:**
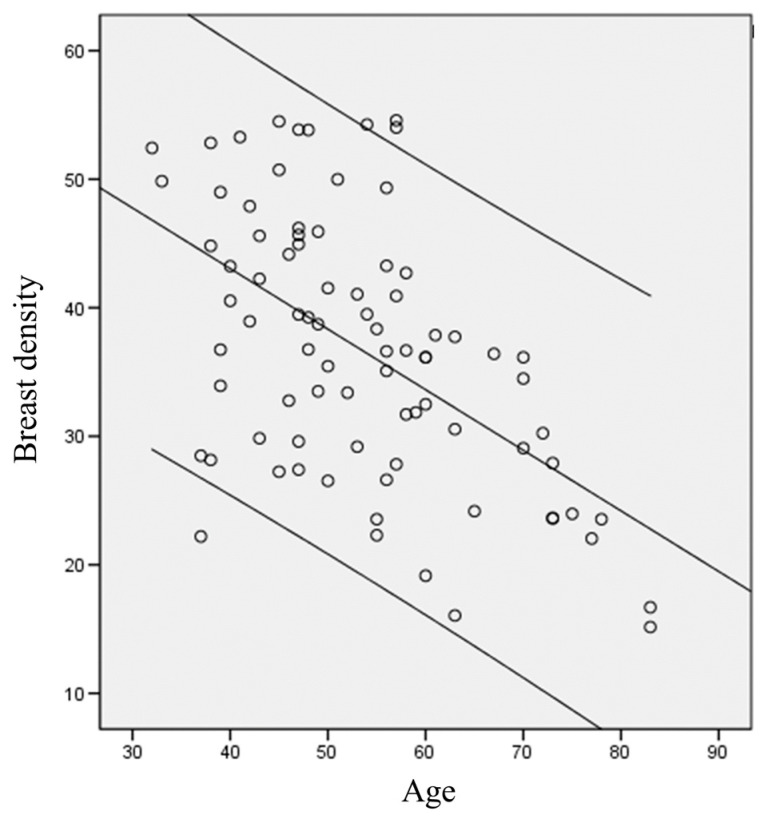
Relationship between age and breast density.

This study used the K-means algorithm to classify breast density. Numerous studies have employed the K-means algorithm in CT or magnetic resonance image analysis [17], as well as artificial intelligence. This study intended to observe whether the performance of mammography was consistent with the visual diagnoses of physicians. The resolution of mammography is extremely high, and mammary glands and fat have clear differences. Therefore, we analyzed each image five times and computed averages to reduce errors. In experiment 2, the breast density of the 84 patients with breast cancer was calculated, and the K-means algorithm was used to match the breast density to the patient’s age. Next, Pearson correlation was used to investigate the linear relationship between age and breast density (Figure 2). The coefficient of correlation between age and breast density was −0.538, which was significantly negative (*p* < 0.01), and Figure 2 shows a clear linear relationship. This result indicated that the breasts of younger patients were denser, and breast density decreased with age.

Another study has also indicated that the breast density of women decreases as they age [20] because female breasts mainly consist of mammary glands and fat. Mammary glands are used to secrete milk and are active at child-bearing ages. Therefore, the breast density of younger women is higher. As women age, their mammary glands degenerate and fat gradually replaces the mammary glands, resulting in a breast density decrease. Breast density is currently clinically determined by a radiological technologist using mammography images because a quantitative standard does not exist. Consequently, human errors occur frequently. This study used mammography images to perform analyses and aimed to accurately classify breast density into different classes.

**Figure 3:**
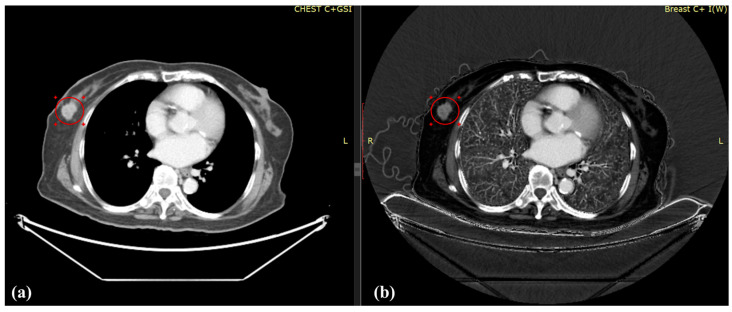
(**a**) Monochromatic spectral image and (**b**) iodine map for a 45-year-old patient with breast cancer and breast density of 54%; the average T/B, T/M, and CNR_b_ were (**a**) 2.68, 1.15, and 7.65, respectively, and (**b**) 7.88, 1.59, and 14.81, respectively.

**Figure 4:**
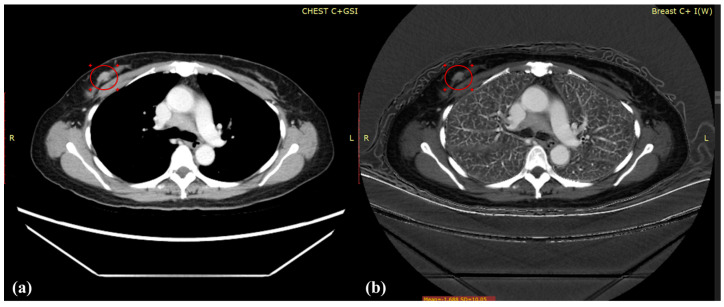
(**a**) Monochromatic spectral image and (**b**) iodine map for a 60-year-old patient with breast cancer and breast density of 19%; the average T/B, T/M, and CNR_b_ was (**a**) 2.62, 1.06, and 6.15, respectively, and (**b**) 4.67, 1.36, and 8.55, respectively.

This study compared the age of the patients with their monochromatic spectral image or iodine map and discovered that the average and maximum T/B, T/M, and CNR_b_ in the monochromatic spectral image and iodine map increased as the breast density increased, as revealed in Table 3, Table 4 and Table 5, respectively. Figure 3 displays a monochromatic spectral image and iodine map for a young patient with high breast density (54%); superior results were obtained using the iodine map. By contrast, Figure 4 displays those of an older patient with low breast density (19%); the results obtained from her iodine map are less clear. Breast density is positively correlated with breast cancer, and mammography cannot provide comprehensive evaluations for the follow-up of young patients with breast cancer; hence, iodine maps can be employed to provide data with sharper contrast to serve as a reference for physicians during diagnosis.

According to a comparion of chest CT using DECT and SECT revealed that the mean CTDI_vol_ is 7.6 ± 0.9 mGy vs. 9.9 ± 1.4 mGy (for patients with BMI ≤ 25 kg/m^2^); 7.7 ± 0.9 mGy vs. 10.8 ± 1.6 mGy (for patients with BMI >25 to <30 kg/m^2^); 9.3 ± 1.6 mGy vs. 13.3 ± 3.7 mGy (for patients with BMI ≥ 30 kg/m^2^). There were significant differences in objective assessments for DECT were 26.8% lower than SECT, but no significant differences in subjective assessments for the image quality [21]. The mean radiation dose in the current study was 6.4 ± 0.6 mGy, which is comparable to that for DECT by Topçuoğlu [21], but is 35% lower than that of SECT for patients with BMI ≤ 25 kg/m^2^. DECT is a promising approach that provides quantitative visualization as well as the ability to suppress material such as iodine while maintaining radiation dose. Dual-energy techniques acquire spectral CT datasets by scanning the same area twice with different kilovolt peaks. This can be achieved either by dual-source CT or through rapid tube potential switching (our study). However, these approaches may require patients to be prospectively selected for DECT. In the latest generation, multiple energy spectral photon counting DECT systems use a dual-layer detector [22,23] to capture the characteristic attenuation of different materials at multiple energy spectrums, with the top row detecting low-energy photons and the bottom layer detecting high-energy photons, this method may provide high detection efficiencies within the human diagnostic energy range (30–120 keV) for breast cancer detection.

## 5. Conclusions

In DECT, monochromatic spectral images are mainly used by physicians for diagnoses, and tissues, such as mammary glands, tumors, fat, and lymph nodes in the images must be clearly distinguished by physicians after comprehensive detection. This study proposed that monochromatic spectral images obtained at 80 keV are the most suitable images for such use. In addition, this study proved that excellent T/B, T/M, and CNR values are achieved for iodine maps when distinguishing normal mammary glands from malignant breast cancer, particularly for breasts of density > 25%. Therefore, using monochromatic spectral images and iodine maps can make the identification of small tumors and distinguishing of tumor edges more straightforward.

## Figures and Tables

**Table 1:** Values of the tumor-to-breast mammary gland ratio (T/B), tumor-to-muscle ratio (T/M), contrast-to-noise ratio of breast tumor (CNR_b_), and contrast-to-noise ratio of metastasized tumor (CNR_m_) for each monochromatic spectral image. The data are shown as the median (minimum value, maximum value).

	40 keV	50 keV	60 keV	70 keV	80 keV	90 keV	100 keV	110 keV	120 keV	130 keV	140 keV	*p* Value
T/B	5.04 (3.23, 12.66)	4.25 (3.00, 10.21)	3.70 (2.76, 8.37)	3.23 (2.54, 7.09)	2.90 (2.33, 6.13)	2.75 (2.15, 5.65)	2.62 (1.97, 5.55)	2.51 (1.83, 5.45)	2.43 (1.73, 5.35)	2.36 (1.66, 5.28)	2.30 (1.59, 5.28)	0.03
T/M	2.30 (1.42, 2.80)	2.01 (1.28, 2.43)	1.76 (1.16, 2.14)	1.54 (1.02, 1.89)	1.37 (0.93, 1.69)	1.25 (0.86, 1.56)	1.18 (0.81, 1.44)	1.12 (0.77, 1.35)	1.09 (0.73, 1.29)	1.05 (0.71, 1.23)	1.03 (0.69, 1.19)	<0.001
CNR_b_	7.39 (4.64, 13.88)	7.02 (4.42, 13.21)	6.60 (4.14, 12.69)	6.11 (3.86, 12.18)	5.72 (3.56, 11.58)	5.30 (3.38, 10.60)	4.85 (3.16, 10.61)	4.49 (3.07, 10.32)	4.26 (2.95, 10.28)	4.08 (2.84, 10.55)	3.94 (2.92, 10.27)	0.02
CNR_m_	39.20 (19.10, 66.24)	54.63 (26.49, 89.59)	69.30 (34.85, 119.00)	88.89 (43.17, 149.96)	105.58 (51.04, 169.54)	119.96 (57.40, 190.95)	131.03 (62.63, 212.71)	136.51 (68.01, 225.32)	150.86 (71.23, 239.46)	158.40 (74.45, 258.75)	164.58 (77.09, 262.74)	<0.001

**Table 2:** Average and maximum T/B, T/M, and CNR_b_ obtained from monochromatic spectral images and iodine maps for all breast densities.

n = 84	Monochromatic Spectral Image	Iodine Map	*p* Value
T/B			
Average value	3.24 ± 2.27	5.08 ± 4.96	0.002
Maximum value	4.61 ± 3.67	7.72 ± 5.18	<0.001
T/M			
Average value	1.21 ± 0.32	2.15 ± 1.02	<0.001
Maximum value	1.36 ± 0.32	2.61 ± 1.05	<0.001
CNR_b_			
Average value	6.90 ± 2.77	10.13 ± 5.24	<0.001
Maximum value	7.92 ± 3.25	11.55 ± 5.69	<0.001

**Table 3:** Comparison of T/B, T/M, and CNR_b_ obtained from the monochromatic spectral images and iodine maps for breast density of 0–24%.

n = 13 0–24%	Monochromatic Spectral Image	Iodine Map	*p* Value
T/B			
Average value	2.53 ± 1.23	4.27 ± 1.74	0.05
Maximum value	2.90 ± 1.29	5.82 ± 3.89	0.03
T/M			
Average value	1.19 ± 0.23	1.83 ± 1.19	0.05
Maximum value	1.33 ± 0.44	2.58 ± 1.86	0.008
CNR_b_			
Average value	6.64 ± 1.95	8.65 ± 3.43	0.01
Maximum value	7.32 ± 2.01	9.63 ± 3.13	0.007

**Table 4:** Comparison of T/B, T/M, and CNR_b_ obtained from the monochromatic spectral images and iodine maps for breast density of 25–49%.

n = 59 25–49%	Monochromatic Spectral Image	Iodine Map	*p* Value
T/B			
Average value	3.36 ± 2.52	4.59 ± 3.19	0.01
Maximum value	4.63 ± 3.47	7.62 ± 3.49	0.006
T/M			
Average value	1.21 ± 0.37	2.19 ± 0.99	0.002
Maximum value	1.36 ± 0.30	2.60 ± 0.69	<0.001
CNR_b_			
Average value	6.69 ± 3.15	10.37 ± 5.45	0.01
Maximum value	7.19 ± 3.25	11.45 ± 6.70	0.01

**Table 5:** Comparison of T/B, T/M, and CNR_b_ obtained from the monochromatic spectral images and iodine maps for breast density of 50–74%.

n = 12 50–74%	Monochromatic Spectral Image	Iodine Map	*p* Value
T/B			
Average value	3.67 ± 1.85	4.46 ± 3.49	0.006
Maximum value	6.12 ± 5.31	9.91 ± 4.14	0.04
T/M			
Average value	1.24 ± 0.32	2.22 ± 0.94	<0.001
Maximum value	1.43 ± 0.27	2.62 ± 0.89	<0.001
CNR_b_			
Average value	7.01 ± 2.88	10.54 ± 5.88	<0.001
Maximum value	8.11 ± 3.46	11.86 ± 5.86	<0.001

## Data Availability

Not applicable.
